# Cell injury after ischemia and reperfusion in the porcine kidney evaluated by radiolabelled microspheres, sestamibi, and lactadherin

**DOI:** 10.1186/2191-219X-3-62

**Published:** 2013-08-07

**Authors:** Stine S Pedersen, Anna K Keller, Marie K Nielsen, Bente Jespersen, Lise Falborg, Jan T Rasmussen, Christian W Heegaard, Michael Rehling

**Affiliations:** 1 Institute of Clinical Medicine, Aarhus University, Aarhus, Denmark; 2 Department of Urology, Aarhus University Hospital, Skejby, Denmark; 3 Department of Nephrology, Aarhus University Hospital, Skejby, Denmark; 4 Department of Clinical Physiology and Nuclear Medicine, Aarhus University Hospital, Skejby, Denmark; 5 Protein Chemistry Laboratory, Department of Molecular Biology and Genetics, Aarhus University, Aarhus, Denmark

**Keywords:** Kidney, Ischemia, Perfusion, Apoptosis, Mitochondria, Lactadherin, Sestamibi, Caspase-3, Glomerular filtration rate

## Abstract

**Background:**

The purpose of the present study was to quantify renal cell injury after ischemia and reperfusion in a pig model using ^99m^Tc-lactadherin as a marker of apoptosis and ^99m^Tc-sestamibi as a marker of mitochondrial dysfunction.

**Methods:**

Thirty-four pigs were randomized into unilateral renal warm ischemia of 120 (WI_120_) or 240 min (WI_240_). The glomerular filtration rate (GFR) was calculated by renal clearance of ^51^Cr-ethylenediaminetetraacetic acid, and apoptosis was quantified by immunohistochemical detection of caspase-3. After 240 min of reperfusion, intravenous ^99m^Tc-lactadherin or ^99m^Tc-sestamibi was injected simultaneously with ^153^Gd microspheres into the aorta. *Ex-vivo* static planar images of the kidneys were acquired for determination of the differential renal function of tracer distribution using a gamma camera.

**Results:**

In WI_120_, there was no significant difference in the uptake of microspheres in the ischemic and contralateral normal kidney indicating adequate perfusion (uptake in ischemic kidney relative to the sum of uptake in both kidneys; 46% ± 12% and 51% ± 5%). In WI_240_, the uptake of microspheres was severely reduced in both groups (17% ± 11% and 27% ± 17%). GFR was severely reduced in the post ischemic kidney in both groups.

In both groups, the uptake of lactadherin was reduced (41% ± 8%, 17% ± 13%) but not different from the uptake of ^153^Gd microspheres. Caspase-3-positive cell profiles were increased in the post-ischemic kidneys (*p* < 0.001) and increased as the length of ischemia increased (*p* = 0.003). In both WI_120_ and WI_240_, the amount of ^99m^Tc-sestamibi in the ischemic kidney was significantly lower than the amount of ^153^Gd microspheres (40 ± 5 versus 51 ± 5 and 20 ± 11 versus 27 ± 17; *p* < 0.05).

**Conclusions:**

In an established pig model with unilateral renal warm ischemia, we found significantly reduced ^99m^Tc-sestamibi uptake relative to perfusion in the kidneys exposed to ischemia indicating a potential ability to detect renal ischemic and reperfusion injuries. However, apoptosis was not detected using ^99m^Tc-lactadherin in the post-ischemic kidneys despite increased number of caspase-3-positive cell profiles.

**Trial registration:**

This study is approved by the Danish Inspectorate of Animal Experiments (2010/561-1837).

## Background

Kidney transplantation is associated with renal ischemia and reperfusion (I-R). I-R may cause acute kidney injury seen as delayed graft function (DGF), which is a frequent complication of renal transplantation. DGF is associated with an increased risk of rejection, probably related to sequelae of I-R injury, but it is a relatively a benign condition in contrast to irreversible graft necrosis that is occasionally seen [1-6]. Graft necrosis should be detected early and the graft removed. Cell death by ischemia may occur by apoptosis as well as necrosis in experimental models of renal injury [7,8].

A clinical or radiological method to diagnose the reversible acute tubular necrosis of DGF from transplant rejection and/or irreversible cell death does not exist, except for percutaneous ultrasonography-guided renal biopsy. This is an invasive procedure, and the severities of histological lesions are not always clear. Ultrasonography imaging of the allograft can be used to assess blood perfusion, but perfusion may be intact for some time despite severe renal tissue necrosis. When prerenal and postrenal causes of a failing graft are excluded, allograft biopsy is needed to determine the state of the tissue. Biopsy may be associated with complications, and therefore, the frequency of the procedure should be kept low [9,10].

There is a need for non-invasive tests monitoring graft function and complications both in clinical routine and in the search for new treatments. In preclinical proof-of-concept studies, imaging techniques are useful for quantification of tissue damage to identify interventions that may translate into clinical benefit for the patient. This is needed also in the growing field of transplantation after circulatory death, where long-term warm ischemia may prevail [11].

Apoptosis seems to play an important role in renal injury caused by I-R [7,9]. The mitochondria exert an important role in apoptosis, i.e., the mitochondrial membrane becomes more permeable leading to the release of potentially toxic proteins. Both apoptosis and changes in mitochondrial membrane permeability may be visualized with radiolabelled tracers. Cells undergoing apoptosis or necrosis can be detected with annexin A5, which is a cellular protein with binding affinity to the phospholipid phosphatidylserine (PS), which is increasingly exposed on the surface of apoptotic and necrotic cells. Recently, ^99m^Tc-labelled lactadherin was introduced as a more sensitive protein for the detection of PS because of a higher affinity [12-15]. ^99m^Tc-labelled sestamibi (MIBI), on the other hand, has been used clinically for quantification of myocardial perfusion in ischemic heart disease for about 25 years. Uptake appears to be passive across the plasma and mitochondrial membranes with the lipophilic cationic MIBI being retained in the mitochondria by the large negative membrane potential [16-18]. It declines to low levels with mitochondrial depolarization as a result of compromised mitochondrial respiration.

In trials performed at our facilities concerning I-R in the porcine myocardium, we observed distinct uptake of lactadherin in the ischemic area (unpublished observations by Poulsen RH, Rasmussen JT, Bøtker HE, Falborg L, Heegaard CW, Rehling M) and a corresponding defect in the uptake of MIBI after normalization of the flow in the former obstructed artery [19]. These observations indicate substantial cell death by either apoptosis or necrosis in the former ischemic area and malfunction of the mitochondria even after normalization of the coronary flow.

The aim of the present study was to quantify reversible and irreversible renal injury after renal I-R in a pig model. Renal cell injury was investigated using ^99m^Tc-lactadherin. Mitochondrial dysfunction was studied using ^99m^Tc-sestamibi, and the histological marker of apoptosis caspase-3 was used on renal tissue.

## Methods

### Study setup

An animal model offers the opportunity to study organs *in vivo* and the porcine model was chosen to simulate a renal transplantation with complications. The study was approved by the Danish Inspectorate of Animal Experiments (2010/561-1837) and performed according with their guidelines.

Thirty-four female Danish Landrace/Yorkshire pigs, weight 38 ± 2 kg, were studied. The pigs were divided into four groups. The pigs were exposed to unilateral renal warm ischemia (WI) of 120 or 240 min (WI_120_ and WI_240_). In both groups, the distribution of either MIBI or lactadherin was investigated in separate groups, as they were marked with the same radionuclide (^99m^Tc). Consequently, WI_120_ = MIBI_120_ + Lact_120_ and WI_240_ = MIBI_240_ + Lact_240_.

Renal ischemia was induced by clamping the renal artery and complete occlusion was verified by the observation of a change in color and tonus. After clamp removal, the kidney was inspected for restoration of blood flow. Side randomization of ischemic kidney (IK) was performed in each pig. The contralateral kidney served as control (CK).

### Animals and anesthesia

**Figure 1:**
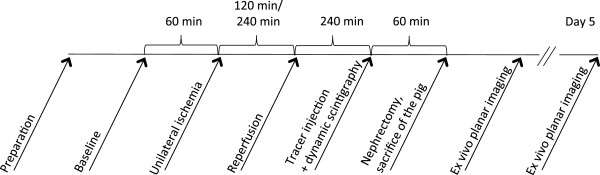
Experimental protocol.

A schematic presentation of the protocol is shown in Figure 1. After overnight fasting, the pigs were sedated with intramuscular injection of midazolam (0.5 mg/kg) before arrival at the facility. Anesthesia was induced with intravenous injection of s-ketamin (5 mg/kg) and midazolam (0.5 mg/kg). The animal was subsequently intubated and mechanically ventilated.

Catheters were inserted in the left jugular vein and carotid artery for continuous monitoring of blood pressure, collection of blood samples and infusion of drugs and fluids. Anesthesia was maintained throughout the experiment with pentobarbital (4 mg/kg/h). The animals received fentanyl 12 μg/kg/h for pain management and were kept hydrated with isotonic saline infusion (12.5 ml/kg/h). They were heparinized with 5,000 IU mixed in the NaCl infusion during the first 3 h. The ventilation was adjusted to maintain a tidal CO_2_ between 4.5 and 5.5 kPa. The animals were placed on heating blankets, and body temperature was monitored with a rectal probe. Every half hour an arterial blood sample was analyzed on an ABL615 apparatus (Radiometer, Copenhagen, Denmark) to monitor the pigs. At the end of the experiment, the animals were put to death with an overdose of pentobarbital while under anesthesia. Each experiment lasted 10 to 12 h.

### Surgical procedures

Under aseptic conditions, both kidneys were retroperitoneal exposed through a midline incision. The ureters were catheterized bilaterally for collection of urine. The renal artery was exposed on one side for clamping. The aorta was catheterized (Launcher® Coronary Guide Catheter, 7 F ID (0.81″), Medtronic, Inc., Minneapolis, MN, USA) through the femoral artery with the catheter tip placed proximally to the renal arteries for the injection of microspheres. At the end of the experiment, biopsies were taken from both kidneys, and bilateral nephrectomy was performed.

### Radiotracer studies

According to randomization, either 300 MBq MIBI or 300 MBq lactadherin was injected into a central vein following 240 min of reperfusion. The purification and preparation of lactadherin was described previously [20]. It was coupled with HYNIC and labelled with ^99m^Tc as reported recently [15]. MIBI was prepared using a commercially available kit of lyophilized tetrakis (2-methoxy isobutyl isonitrile) (Cardiolite; Bristol-Myers Squibb, Belgium). This was labelled according to the manufacturer’s instructions by adding 10,000 MBq of freshly eluted ^99m^Tc (as pertechnetate) to a final volume of 4 ml with saline. Radiochemical purity was above 95%.

### Perfusion

Renal perfusion was determined using a radiolabelled microsphere technique. Simultaneously with the tracer injection, 15 to 20 MBq ^153^Gadolinium-labelled microspheres (15 μm in diameter, NEN-TRAC GD 1560 Microspheres; PerkinElmer, Waltham, MA, USA) were injected in aorta 10 cm above the renal arteries. The diameter of these microspheres was larger than that of a normal capillary bed, allowing them to be trapped by the microcirculation after arterial injection. The microspheres were distributed in the kidneys according to regional blood flow. Counting microsphere activities in the two kidneys on a gamma camera and comparing counts in the two kidneys determined the flow distribution.

### Dynamic and static planar imaging

Renal imaging was performed using a gamma camera (BrightView, Philips Healthcare, Andover, MA, USA) with a low-energy high-resolution collimator. After tracer injection, a dynamic scintigraphy of the abdomen in the posterior position was performed. The dynamic scan was 1 h of duration with 60 frames of 10 s followed by 50 frames of 60 s.

We acquired *ex vivo* static planar images of the kidneys for determination of the tracers distribution between the two kidneys. Planar images were obtained in a 256 × 256 acquisition matrix. All static images were counted to 1 M counts to achieve the same statistic in the images. The acquisition duration is therefore a measurement of the quantity of isotope in the tissue. For ^99m^Technetium, the energy window used were 140 keV ± 10%. When counting ^153^Gadolinium, we used two windows; 35.3 to 47.7 and 90.0 to 110.0 keV as ^153^Gd has several photopeaks. Counts from both windows were added to reach the 1 M counts. ^153^Gadolinium has a half-life longer than that of ^99m^Technetium. This was utilized when obtaining the planar images. The counting of Technetium-labelled tracers was performed directly after nephrectomy. The imaging procedure was repeated 5 days later for detection of ^153^Gadolinium-labelled microspheres after ^99m^Technetium had decayed. The kidneys were stored in refrigerator in the mean time. The differential renal function (DRF) was calculated using the standard software on a Philips JetStream workstation (Philips Healthcare).

The uptake rate of a tracer in the kidney (J) can be calculated from the equation: J = RBF × E × Ca, where RBF is renal blood flow, E the renal extraction fraction of the tracer, and Ca the arterial blood concentration. When comparing the tracer uptake in the right and left kidneys, Ca is cancelled out because the Ca is the same in blood to the two kidneys. Consequently, the equation is reduced to RBF × E, which equals the renal clearance (Clearance = RBF × E). The DRF reflects the individual kidney clearance relative the sum of clearance in the two kidneys. When comparing the relative uptake of two tracers at the same time, the RBF is cancelled out. The comparison therefore reflects relative renal tracer extraction E. When we compare the DRF of MIBI or lactadherin with the distribution of microspheres, we will achieve the same DRF if the extraction of the tracer is the same in the right and left kidneys. In case of a difference in the DRF, we do not know whether this is due to a lower extraction in one kidney or a higher extraction in the other kidney.

### Glomerular filtration rate

Individual kidney glomerular filtration rate (GFR) was measured by a continuous infusion urinary clearance technique using ^51^Cr-Ethylenediaminetetraacetic acid (EDTA). Subsequent to operating procedures, the pigs received 3.2 MBq ^51^Cr-EDTA as a bolus (Behring, Marburg, Germany). It was followed by a continuous infusion of 1.6 MBq/h of ^51^Cr-EDTA during the entire experiment.

Urine was sampled continuously from both ureters and plasma samples were collected hourly. All samples were counted in a gamma counter (Packard Cobra II, GMI Inc., Clearwater, MN, USA) to a statistical accuracy of 1%. The recorded counts were corrected for radioactive background and decay.

### Immunohistochemistry

Straight before nephrectomy, two biopsies of the renal cortex were taken from the upper pole of each kidney using an 8-mm biopsy punch. The biopsies were preserved in 4% formalin for 8 h and stored in phosphate buffer before they were embedded in paraffin and sectioned (2 μm). Immunohistochemical detection of caspase-3 active cell profiles was performed to quantify the degree of apoptosis.

Sections were deparaffined, rehydrated, and antigen unmasked in citrate buffer. Blocking for endogen peroxidase and unspecific binding was performed. Tris-buffered saline was used as wash buffer. Indirect immunohistochemistry was carried out using EnVision Flex+/horse-radish peroxidase as amplification system with DAB + cromogen as substrate. For location and semi-quantification of apoptosis, polyclonal anti-active caspase-3 IgG (Abcam, Ab13847) was used as the primary antibody. Rabbit linker and Envision FLEX + (Dako, K8024) were added. For positive controls, abdominal lymph nodes were used. Negative controls were run without primary antibody incubation.

### Caspase-3 quantification

Quantification of caspase-3-positive cell profiles was performed using the two arbitrary sections from each animal. An unbiased counting frame was used, and counting was performed using an × 20 lens at a total magnification of × 794. The fraction of caspase-3-positive cell profiles was calculated from the following formula:

$$N_{A}=\frac{\mathrm{\Sigma Q}}{\mathrm{\Sigma A}}=\frac{\Sigma Q_{\left(\mathrm{caspase}-3\right)}}{a_{\left(mm^{2}\right)}x\;\mathrm{\Sigma P}},$$

in which *Q* was the number of caspase-3-positive cell profiles, *a* was the area of the counting frame, and *P* was the number of counting frames evaluated.

### Statistics

Data are presented as mean ± SD or median (interquartile range) as appropriate. Statistical analyses were performed with SigmaStat 3.5 (Systat Software, Inc., Richmond, VA, USA).

Comparisons between control and experimental kidneys were established by paired two-tailed *t* test for normally distributed (tracer distribution) or the Wilcoxon signed rank for nonparametric values (caspase-3). The same was used for the increase in GFR (paired two-tailed *t* test) and diuresis (Wilcoxon signed rank) in the control groups compared to baseline. Differences between the groups were established by unpaired two-tailed *t* test values (GFR) or by Mann–Whitney rank sum test (caspase-3). *p* < 0.05 were considered statistically significant.

## Results and discussion

### Results

Thirty-four pigs were randomized and went through the study. One pig from Lact_120_ died directly after tracer injection at the end of the reperfusion phase (*t* = 420) from unknown causes, and for this reason was excluded from the study. Baseline parameters are presented in Table 1.

**Table 1:** Baseline parameters for groups with warm ischemia (WI) of 120 and 240 min

**Parameter**	**Mean ± SD**	
	**WI**_**120**_	**WI**_**240**_
Weight (kg)	38 ± 1.2	38 ± 1.0
MAP (mmHg)	74 ± 11^*^	86 ± 20^*^
HR (beats/min)	50 ± 9	50 ± 10
pH	7.45 ± 0.03	7.44 ± 0.05
Lactate (mmol/l)	1.15 ± 0.48	0.97 ± 0.36

Data are presented as mean ± SD. Groups were compared using unpaired two-tailed *t* test. ^*^p = 0.04.

#### GFR (^51^Cr-EDTA) and diuresis

**Figure 2:**
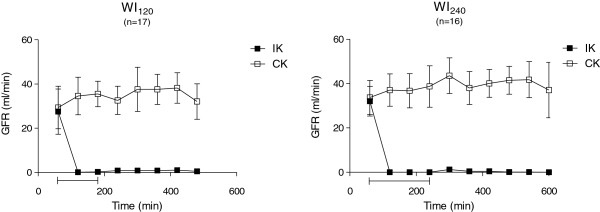
**GFR for groups with warm ischemia (WI) of 120 (left) and 240 (right) min.** Data are presented as mean ± SD. Capped line indicates ischemic period. IK, ischemic kidney; CK, control kidney.

The GFR values from the ischemic kidney and the control kidney before, during, and after ischemia are shown in Figure 2.

**Figure 3:**
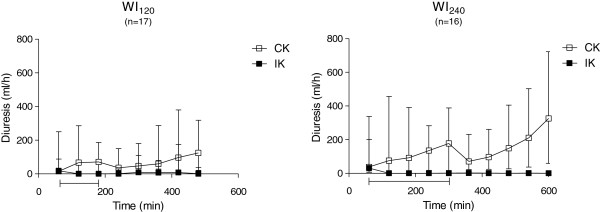
**Diuresis for groups with warm ischemia (WI) of 120 (left) and 240 (right) min.** Data are presented as median (range). Capped line indicates ischemic period. IK, ischemic kidney; CK, control kidney.

At baseline (t_60_), we found a mean single kidney GFR value of 30.2 ± 8.8 ml/min with no significant difference between WI_120_ and WI_240_ or between the ischemic and the control kidney (*p* > 0.1). Immediately after initiating unilateral ischemia, the GFR dropped to values near zero in the ischemic kidneys as no urine was produced. During reperfusion, some urine production was established, but GFR stayed close to zero. Figure 3 shows the diuresis during WI_120_ and WI_240_ from the ischemic as well as the control kidneys. In the control kidneys, we found an increase in GFR during the ischemic period which was significant compared to baseline in all but two measurements in each group (WI_120_; t_240_ 32.6 ± 6.4 ml/min (*p* = 0.1), t_480_ 32.1 ± 8.1 ml/min (*p* = 0.3), WI_240_; t_180_ 36.8 ± 7.7 ml/min (*p* = 0.2), t_600_ 37.1 ± 12.5 ml/min (*p* = 0.3)). Compared to baseline (WI_120_ 15 (8,80) ml, WI_240_ 38 (16,121) ml), an increased urine production was observed in the control kidneys during the study period. This was significant in most measurements; however, a few were non-significant, (WI_120_ t_240_, t_300_; WI_240_ t_180_, t_240_, t_360_, t_420_ (*p* > 0.05).

#### Caspase-3

**Figure 4:**
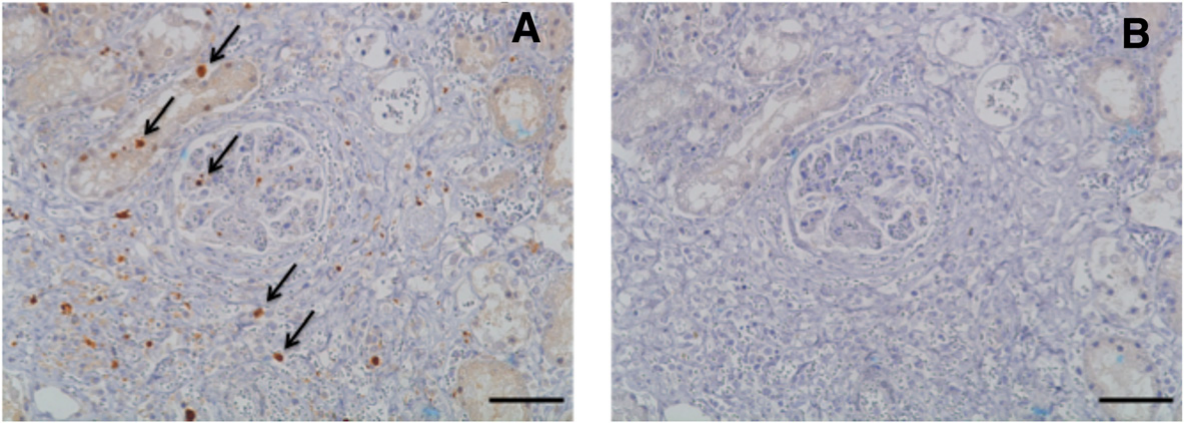
**Representative photomicrographs showing porcine renal cortex. (A)** Active caspase-3-positive IHC staining. Arrows show apoptotic cell profiles in glomeruli, tubules and the lumen of tubules. **(B)** Negative control. Scale bar = 100 μm.

**Figure 5:**
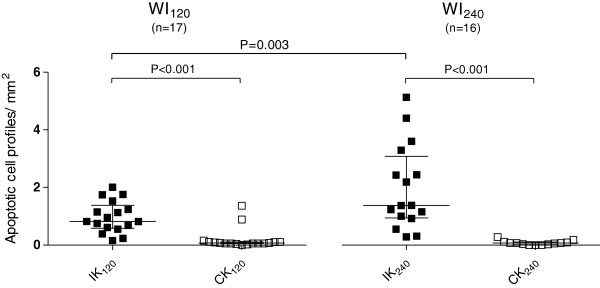
**Apoptosis quantification.** The mean value of apoptotic cell profiles per square millimeter in the two arbitrary sections from each kidney is used. Black horizontal lines are medians, and whiskers are interquartile range. Within the groups, *p* values from Wilcoxon signed rank test are shown, and between the groups *p* values from Mann–Whitney rank sum test are shown. IK, ischemic kidney; CK, control kidney.

A representative picture of the caspase-3-positive staining used for apoptotic cell counts is shown in Figure 4. For quantification, a mean of 104 randomly selected non-overlapping fields of view were used per section. The majority of the apoptotic cells were located in the tubules, but the apoptotic cells were also found in the glomeruli and in the lumen of the tubules. Quantification of caspase-3-positive cell profiles is presented in Figure 5. The degree of caspase-3-active cell profiles presented as median (IQR) were significantly higher in the kidneys subjected to ischemia (IK_120_ 0.86 (0.47,1.36); IK_240_ 1.56 (0.83,2.89)) compared to the control kidney (CK_120_ 0.07 (0.04,0.13), CK_240_ 0.06 (0.00,0.11)), indicating a higher degree of ongoing apoptosis in the affected organ. We found a significant higher degree of apoptotic cell profiles in the sections from the post ischemic kidneys in WI_240_ compared to WI_120_. All these differences were statistically significant (*p* < 0.05).

#### Dynamic and static planar imaging

**Figure 6:**
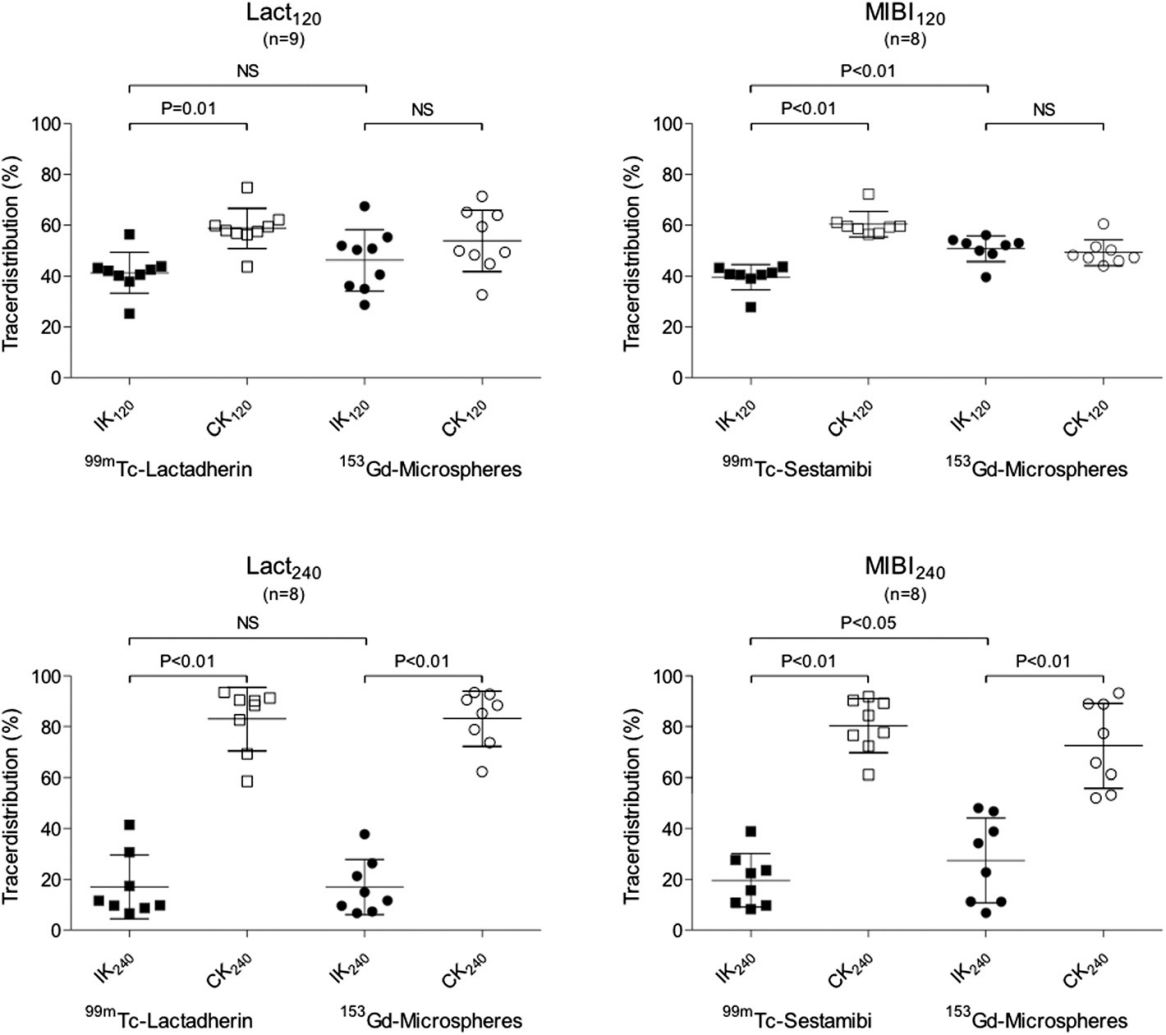
**Tracer distribution in the four groups.** Black horizontal lines are means, and whiskers are SDs. *p* values from paired *t* tests are shown. IK, ischemic kidney; CK, control kidney.

**Figure 7:**
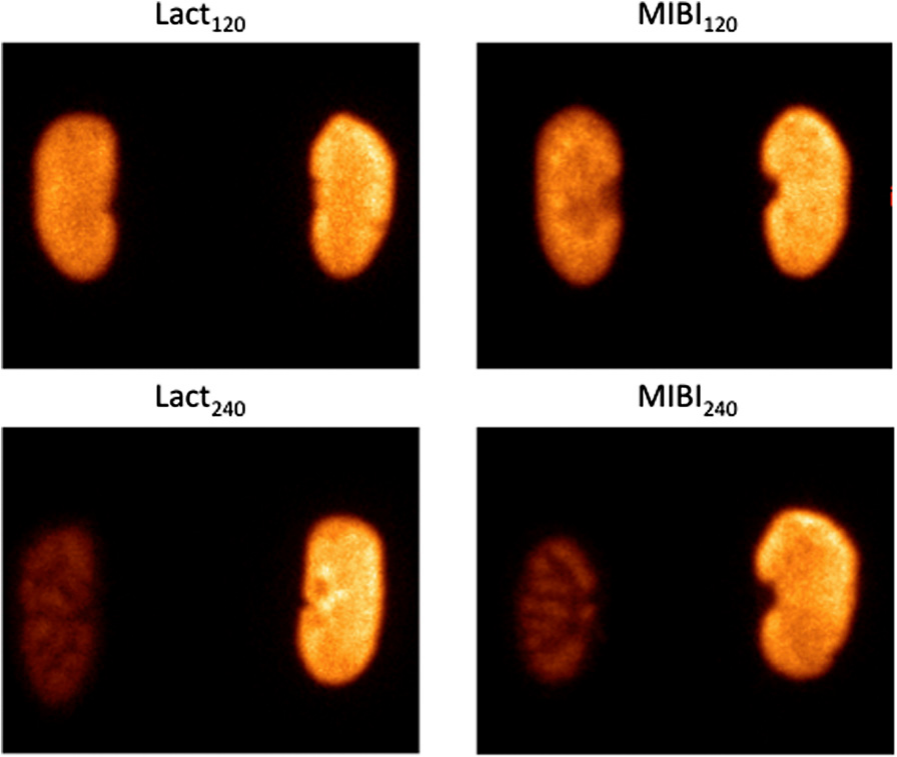
**Representative picture showing *ex vivo *planar imaging of **^**99m**^**Tc-lactadherin and **^**99m**^**Tc-MIBI uptake in four groups.** Post-ischemic kidney (left) and the contralateral kidney (right).

The 60-min dynamic scintigraphy showed an initial renal uptake of the MIBI and lactadherin followed by a steady-state concentration (data not shown). In this study, we did not find any urinary excretion of neither MIBI nor lactadherin. The relative uptake and distribution of the three tracers is shown in Figure 6. The results are presented as fraction of counts in the ischemic kidney relative to the sum of counts in both kidneys (DRF) in percentages (mean ± SD). Figure 7 shows a representative picture of the *ex vivo* planar imaging of ^99m^Tc-Lactadherin and ^99m^Tc-MIBI.

##### Microspheres

The acquisition duration was of 98 (±30) min. In WI_120,_ there was no significant difference in the uptake of microspheres in the ischemic kidney and the contralateral normal kidney neither in the lactadherin group (46 ± 12) nor in the MIBI group (51 ± 5). In WI_240_, the uptake of microspheres was severely reduced both in the lactadherin group (17 ± 11) and in the MIBI group (27 ± 17), both *p* < 0.01.

##### Lactadherin

Duration of acquisition was 42 (±10) min. In WI_120_, the uptake of lactadherin was reduced (41 ± 8) but not different from the normal uptake of microspheres in the same group (46 ± 12). In WI_240_, the uptake lactadherin was severely reduced (17 ± 13) but still not different from the distribution of microspheres (17 ± 11).

##### MIBI

Duration of acquisition was 15 (±5) min. In both WI_120_ and WI_240_, the amount of MIBI in the ischemic kidney was lower than the amount of microspheres (40 ± 5 versus 51 ± 5 and 20 ± 11 versus 27 ± 17), and the differences found were significant (*p* < 0.05).

## Discussion

In the present setup, we studied renal perfusion, GFR, apoptosis, and mitochondrial function in pigs after 120 and 240 min of unilateral renal artery occlusion followed by reperfusion for 300 min. We successfully managed to establish a model with unilateral acute kidney injury seen as low urine production and GFR as well as increased apoptosis quantified with detection of caspase-3-active cell profiles in the kidneys subjected to ischemia. We found no difference in blood flow in the post-ischemic kidney and the control kidney after 120 min of ischemia, but after 240 min, the renal blood perfusion was reduced in the post-ischemic kidney. We did not find any difference in relative uptake of lactadherin compared to microspheres, whereas the relative uptake of MIBI was lower than that for microspheres in the ischemic kidney after both 120 and 240 min of ischemia. This indicates that the renal extraction of MIBI was lower in the ischemic kidney than in the contralateral kidney.

Ischemic acute tubular necrosis (ATN) is by far the most common cause of DGF. Ischemic ATN is caused by prolonged renal hypoperfusion leading to energy depletion and impaired homeostasis. Ischemia starves the tissue from oxygen and nutrients and causes accumulation of metabolic waste products and prolonged ischemia, and the following reperfusion may result in reversible as well as irreversible damage and cellular death. The return of oxygen to the tissue after reestablishment of blood perfusion results in generation of reactive oxygen species. Reactive free radicals cause cell membrane damage and oxidation of membrane proteins and DNA leading to increased cell membrane permeability and enzymatic disturbances [3,6,20]. Despite the term ATN, actual necrosis of tubular epithelium cells is a less common finding than cellular injury and dysfunction. These forms of cell injury can include apoptosis, loss of cell-forming gaps in the tubular architecture and denuded basement membranes, and cells sloughing into tubular lumens [9]. In animal models, luminal hyaline cast formations are seen, which can cause obstruction and dilation of the tubules [9,21,22].

Established ATN is associated with a decreased renal blood flow and subsequently reduced GFR and even anuria [23]. In WI_120_, an equal DRF of microspheres was demonstrated indicating an equal blood flow to the two kidneys, whereas we found a markedly reduced uptake of microspheres in the kidneys subjected to 240 min of ischemia indicating a decreased blood flow in the post-ischemic kidney. However, despite an equal blood flow in WI_120_, GFR stayed close to zero in the entire reperfusion period, indicating a poor filtration fraction in the kidneys subjected to ischemia or simply reabsorption of the tracer due to decreased effective renal blood flow. We observed the pigs in a total reperfusion phase of 300 min (240 + 60 min scintigraphy). As seen in other setups, the kidneys in WI_120_ could regain both GFR and urine production if the reperfusion period was extended [2,24]. On the other hand, an insult of 240 min of WI might be too extreme, bringing the kidneys to a point of no return concerning reestablishment of normal function as indicated by the reduced blood flow and the more pronounced apoptosis in the present study, although a longer observation time would be necessary to confirm this [22,25].

MIBI is delivered to the tissue in proportion to the regional blood flow and, due to transmembrane potentials, retained within the mitochondria. However, as MIBI is lacking a chemical microsphere behavior, intracellular retention is depended on sustained transmembrane potentials. Therefore, alterations of mitochondrial function caused by severe ischemia may reduce the accumulation of MIBI within the cells [26]. Ischemic injury results in the entering of extracellular Ca^2+^ in the cytosol, which is rapidly sequestered in the mitochondria as well as other organelles resulting in mitochondrial destruction. Thus, the uptake and retention of MIBI is inhibited, and MIBI is therefore hypothesized to be a highly sensitive marker of cell viability [18]. This has been observed clinically in patients with reperfused myocardial infarcts, where the absence of MIBI correlates with the lack of return of myocardial function [27].

Accordingly, we found that the retention of MIBI was reduced in the ischemic kidneys in both groups compared to the contralateral kidney. This equalled the amount taken up, as no MIBI was found in the urine. In both MIBI_120_ and MIBI_240_, the MIBI uptake was less than the microsphere uptake in the post-ischemic kidney indicating a relative lower renal extraction of MIBI in the post-ischemic kidney compared to the contralateral kidney. This indicates that MIBI might be able to detect renal damages as diminished mitochondrial function after I-R insults. However, our results do not show, whether renal damage indicated by reduced MIBI uptake is reversible or irreversible, which could be of very high interest, e.g., in the renal transplantation setting. The relative uptake of MIBI and microspheres was performed in the same group of pigs as paired observations. We did not plan to compare uptake of MIBI with uptake of microspheres in the lactadherin group. In small groups with variation in between the pigs, the risk of false negative results is high when using non-paired observations.

Lactadherin binds to the specific surface phospholipid PS, which is exposed on the cell surface during apoptosis or early necrosis and facilitates phagocytosis by acting as a bridge between exposed PS and the avβ3 integrin on the macrophage surface [28-32]. In contrast to the widely used and structurally unrelated PS-binding protein annexin V, lactadherin binding appears proportional to the PS content and is independent of Ca^2+^ concentration and membrane phosphatidylethanolamine content. Lactadherin has been shown to be more sensitive in detecting PS exposure than annexin V and during apoptosis lactadherin can detect PS-expressing apoptotic cells earlier than annexin V [13]. Furthermore, annexin V is excreted through the kidneys and is therefore not suitable for detecting renal apoptosis.

We hypothesized, that the accumulation of lactadherin would be greatest in the kidneys subjected to ischemia, as a result of increased cell death. Apoptosis was present in both tubular and glomerular cells as well as in the tubule lumen quantified with immunohistochemistry detection of caspase-3. Despite this, we found a significantly decreased uptake of lactadherin in the post-ischemic kidney compared to the contralateral non-ischemic kidney. We did not find any difference between the relative uptake of lactadherin and microspheres, indicating that the distribution of lactadherin may simply follow blood supply. Accordingly, the results of this study indicate that the current setup does not allow lactadherin to function as a non-invasive marker of neither ongoing apoptosis nor necrosis in the kidneys after the herein chosen I-R insults.

The Danish Landrace pigs were chosen as the experimental model as the porcine kidney is multipapillate and has calyces similar to the human kidney as well as physiologic parameters comparable to humans [33,34]. Historically, 30 min of renal WI have been considered the upper limit for allowing complete recovery of renal function in humans. However, studies concerning WI in porcine kidneys have demonstrated that a WI of up to 120 min results in full recovery of renal function [24].

By use of an extended WI (120 and 240 min), a relevant model of donation after circulatory death was made; furthermore, it was ensured that sufficient unilateral renal damage occurred in the healthy pigs studied. Most cases of ischemic ATN occur in patients with predisposing comorbidities in sharp contrast to the healthy animals used in experimental setups. Thus, when setting up an animal model, intense insults may be required to achieve reproducible experimental renal failure. Of course, it is a weakness that the apoptosis and lack of filtration caused by the I-R injuries were not observed on the long term in the present study since I-R injuries may heal with time in kidneys with acute tubular necrosis [23,24,35].

The tracers were injected after 4 h of reperfusion, allowing ischemia as well as reperfusion injury to occur. The pigs were sacrificed 60 min after tracer administration, which allowed sufficient tracer uptake from the bloodstream [36]. ^153^Gadolinium-labelled microspheres are removed from the circulation in the first-pass phase of the circulation as the spheres are trapped within the microcirculation after arterial injection. In patients, the arterial blood clearance of ^99m^Tc-sestamibi is close to the cardiac output [27,37,38]. Accordingly, the plasma concentration decreases very fast with a blood concentration of less than 1% of peak concentration 3 min after intravenous injection. Biodistribution studies have shown that ^99m^Tc-MIBI is cleared mainly in the liver, muscles, and kidneys [27,37]. The high renal uptake of MIBI and a good counting statistic in a normal kidney is an advantage when detecting reduced uptake of MIBI after ischemia and reperfusion. The results of the present study support the use of MIBI as an agent for measurement of renal injury after I-R. However, further studies will be necessary to clarify whether this demonstrated renal damage is reversible or irreversible.

^99m^Tc-lactadherin is mainly taken up in the liver and the renal extraction of ^99m^Tc-lactadherin was approximately zero 0 to 240 min after intravenous injection [36,39]. The low uptake of ^99m^Tc-lactadherin in normal kidneys is optimal when looking for increased uptake in the entire kidney or as hot spots. Lactadherin has previously been hypothesized to be a new potential tracer for the visualizing of cell death in the kidney [39,40], but our results do not support this. We did only find a high degree of caspase-3-active cell profiles in a few of the ischemic kidneys. It is possible that the extent of apoptosis is not sufficient to be seen on top of the background levels of lactadherin. Even the uptake of lactadherin in the normal kidney is lower than the uptake of annexin V, we cannot exclude that the uptake of lactadherin in the normal kidney reduces the sensitivity in detecting apoptosis in the ischemic kidney.

We found neither lactadherin nor MIBI in the urine after ischemia and reperfusion. In normal mice and pigs, we found that less than 5% of the injected lactadherin was taken by the kidneys, and the renal extraction was close to 0 [36,39]. This may be less in humans, especially after ischemia and reperfusion. MIBI, however, is normally excreted in the urine, and as much as 40% of injected dose has been reported found in the urine 1 h after injection [41]. MIBI may not be excreted in the ischemic damaged kidney, but we have no explanation for the lack of excretion in the contralateral kidney.

## Conclusions

To our knowledge, a study of the present tracers as agents of detecting renal damage after I-R insults has not previously been carried out. In conclusion, in a porcine model with 120 and 240 min of unilateral warm renal ischemia followed by a total of 300 min of reperfusion, apoptosis was visualized in the post-ischemic kidney using caspase-3 immunohistochemistry, but neither apoptosis nor cell necrosis was visualized using radiolabelled lactadherin. We found a reduced ^99m^Tc-MIBI uptake relative to perfusion in the kidneys exposed to ischemia indicating that MIBI might be able to detect mitochondrial dysfunction after renal ischemia and reperfusion. Further studies are needed to clarify the abilities of MIBI as a non-invasive diagnostic tool of renal injuries.

## Competing interests

The authors declare that they have no competing interests.

## Authors’ contributions

All authors contributed substantially to the scientific process leading to this manuscript. Authors SSP, AKK, BJ, and MR contributed to the concept and design of the study. SSP, AKK, and MKN acquired data on the subjects. SSP, AKK, LF, JTR, CWH, and MR analyzed and interpreted the data. SSP, AKK, MKN, and MR drafted the manuscript, which was revised by BJ, LF, JTR, and CWR. All authors read and approved the final manuscript.
